# Unraveling the Interplay: Exploring the Links Between Gut Microbiota, Obesity, and Psychological Outcomes

**DOI:** 10.7759/cureus.49271

**Published:** 2023-11-23

**Authors:** Divya Saravanan, Suhana Khatoon B, Jefry Winner G

**Affiliations:** 1 School of Public Health, SRM Institute of Science and Technology, Chengalpattu, IND; 2 Pharmacology and Therapeutics, Jawaharlal Institute of Postgraduate Medical Education and Research, Pondicherry, IND

**Keywords:** food and nutrition, enteric nervous system disorders, gut-brain axis, microbial therapy, nutrition and metabolism

## Abstract

This narrative review delves into the complex and intricate mechanisms of the gut-brain axis. Gut microbiota has gained immense importance in the treatment of various diseases. The therapeutic potential of gut-microbial modulation is slowly coming to light. With good preclinical evidence, some human studies shed light on the translation potential of gut-microbial modulation. The concept of gut-microbial modulation has been studied for over a few decades. The relationship between gut microbiota and various homeostatic mechanisms is fascinating. Over the years, we have started understanding the immense role of gut microbiota in various homeostatic mechanisms. There are a good number of clinical studies that have shown the therapeutic potential of gut-microbial modulation in obesity and psychological diseases, especially depression and anxiety. The gut-microbial modulation can be achieved by dietary factors or supplementation. In this review, we explore the mechanisms by which prebiotics, probiotics, and synbiotics alter the gut-brain axis. The review limits its discussion to the most recent clinical studies that have shown promise as therapeutic strategies.

## Introduction and background

Gut microbial factors have gained immense importance over the past years. The role of gut microbiota and its alteration in various disease states is being increasingly studied. Multiple strategies can modulate gut microbiota. The strategy for modulation ranges from introducing microbiota directly into the gut to enhancing them with the help of probiotics, prebiotics, and synbiotics.

In recent years, gut-microbial modulation has become effective in various disease conditions. Some conditions worth mentioning are irritable bowel disease, pseudomembranous enterocolitis, and inflammatory bowel disease. In irritable bowel syndrome, the role of gut microbial alterations following various insults has almost been proven [1]. Similarly, fecal microbiota transplantation (FMT) is the latest approved therapy for antibiotic-refractory pseudomembranous enterocolitis [2].

Gut-microbial modulation has emerged as a potential therapeutic approach for treating obesity, considering its significant role in the condition. In the context of obesity, our gut hosts a diverse microflora, with Firmicutes (e.g., Lactobacillus) and Bacteroidetes (e.g., Bacteroides) comprising approximately 90% of the gut microbiota. Notably, individuals with obesity often exhibit a higher proportion of Firmicutes and a diminished population of Bacteroidetes, as indicated by recent studies [3]. This microbial imbalance can contribute to increased energy extraction from food and lead to alterations in host metabolism, ultimately resulting in weight gain [4].

Moreover, the influence of gut microbiota extends beyond the realm of physical health, reaching into the modulation of psychological outcomes in mental health disorders, particularly depression and anxiety. Observations regarding the role of gut microbiota in mental health reveal a bidirectional communication pathway between the gut and the brain through the enteric nervous system (ENS). This intricate gut-brain neural connection influences emotions and behavior, shedding light on the potential interplay between gut microbial composition and mental well-being [5,6].

Gut microbiota modulates this neural communication network with the gut, which can impact emotional responses and potentially serve as a therapeutic target for conditions like autism spectrum disorder [7]. This evidence from the literature has shed light on the potential role of gut-microbial modulation as a therapeutic strategy in obesity and mental health disorders. In this review, we have explained the mechanisms behind complex gut-microbial connections and their implications for obesity, depression, and anxiety. We also have explained the therapeutic potential of prebiotics, probiotics, and synbiotics in treating obesity, depression, and anxiety with evidence from clinical studies.

## Review

Gut-brain axis: Communication between the gut and brain

The gut-brain axis (GBA) is a complex and bidirectional communication system between the gastrointestinal and central nervous systems. In this complex network, various components interact dynamically, including the gut microbiota, ENS, autonomic nervous system (ANS), and hypothalamic-pituitary-adrenal axis (HPA) [8]. The GBA has gained significant attention recently because of its potential role in several neurological and psychological disorders, as well as its far-reaching implications for human health [9].

**Figure 1:**
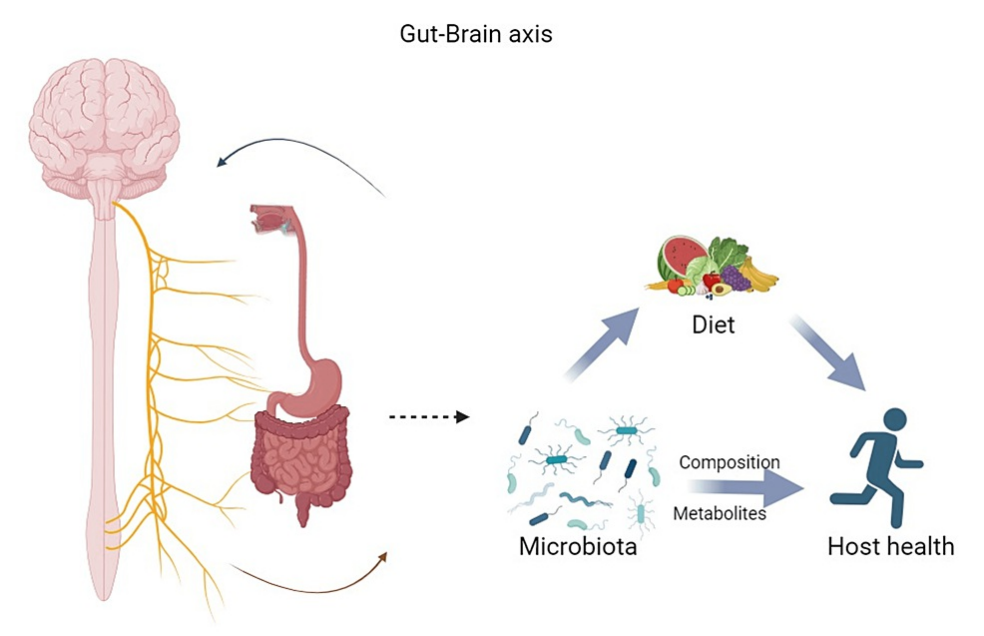
Gut-brain axis An illustration to show the interactions between the gut and brain, created by adapting from [12-13].

Among the most important components of GBA communication is the gut microbiota, which is a complex community of trillions of microorganisms. These microorganisms produce several bioactive compounds, including neurotransmitters and metabolites, which can influence neural function and behavior [10]. An ENS is a network of millions of neurons found in the gut. ENS acts as a local control system to regulate gastrointestinal functions. ENS simultaneously communicates with the central nervous system. Because of the brain’s instructions and the information received from the gut, the ANS, consisting of the sympathetic and parasympathetic branches, modulates the physiological functions of the gut. Furthermore, gut-derived signals can activate the HPA axis, a vital component of the body’s stress response, impacting cognitive and emotional functions (Figure 1) [11].

Influence of gut microbiota on psychological well-being

The connection between gut microbiota and psychological outcomes is a burgeoning field of research with far-reaching implications for mental health. Several factors within the GBA influence psychological well-being. These include gut-brain neural connections, the immune system, and the endocrine system, each with a unique role in shaping psychological outcomes. The interplay between gut microbiota and psychological effects is a dynamic and evolving field of study with significant implications for mental health.

Gut-brain neural connections involving the vagus nerve and ANS create a bidirectional communication pathway that influences emotions and behavior [5,6]. The gut microbiota further modulates this neural dialog, which can impact emotional responses and potentially serve as a therapeutic target for conditions such as autism spectrum disorder [7].

The immune system within the gut plays a pivotal role in shaping psychological outcomes. It is a crucial communication network between the brain and the gastrointestinal tract [14]. Studies on germ-free (GF) animal models underscore how structural and cellular immune deficiencies can profoundly affect psychological well-being [15]. Additionally, the immune system’s involvement in obesity and its association with neuroinflammatory disorders further emphasizes the interconnectedness between the immune system and psychological health [16].

The endocrine system plays a significant role in the GBA, with the gut microbiota modulating the neuroendocrine system and influencing behaviors related to stress, learning, memory, eating, and obesity [17]. Although the neuroendocrine system remains incompletely understood, factors such as hormonal secretion and bacterial metabolites, including short-chain fatty acids (SCFAs) and neurotransmitters, are believed to be pivotal in shaping psychological and behavioral changes [17,18]. The intricate network of connections within the GBA underscores the profound impact of gut microbiota on psychological outcomes and offers promising avenues for mental health research and intervention.

Most of our understanding comes from preclinical studies. Sudo et al. studied whether postnatal microbial colonization could affect the development of brain plasticity and subsequent physiological response. The study evaluated the HPA axis reaction to stress by comparing GF, specific pathogen-free (SPF), and gnotobiotic mice. The study revealed colonizing microbes altered the HPA response to restraint stress, indicating that the interaction of gut bacteria with the brain is also bidirectional, just like the brain-gut axis. This was the first report to show commensal microbes affecting the neural network responsible for controlling stress responsiveness. This study revealed stress response to restraint stress is affected by the limbic system, including the prefrontal cortex, hippocampus, and amygdala, and requires assembly and processing of signals from multiple sensory modalities before initiation of a stress response, giving us a better understanding of the pathways involved [19].

In preclinical studies, oral administration of a single, unique bacterium (*Campylobacter jejuni*) to rats in subclinical doses leads to anxiety. Later, further research conducted in these animals revealed that gut-microbial modulation caused anxiety-like behavior in mice, with concomitant activation of neuronal regions in the brain that were dependent on information received from the gut via the vagus nerve. This research suggested the interaction of ENS in the development of anxiety [20].

Microbiota and obesity

Obesity, a global health epidemic, is a multifactorial condition influenced by genetic, environmental, and lifestyle factors. Emerging research has highlighted the crucial role of the human gut microbiota in the development and progression of obesity [21]. The gut microbiota plays an intricate role in energy metabolism, appetite regulation, inflammation, and energy extraction from dietary sources, which are vital obesity-associated factors [22]. Dysbiosis, an imbalance or alteration in the composition of the gut microbiota, has been implicated in the pathophysiology of obesity. A higher abundance of Firmicutes and a reduced population of Bacteroidetes are common characteristics of obese individuals. These microbial imbalances can lead to increased energy harvest from food and alterations in host metabolism, resulting in weight gain [4].

Moreover, gut microbiota can affect host metabolism by producing SCFAs. The microbiota-driven endocrine pathways can influence appetite regulation and energy expenditure [23]. Leptin and ghrelin, hormones involved in hunger and satiety, are controlled by gut microbial communities [24]. Microbiota-derived metabolites, such as neurotransmitters and neuropeptides, can influence the GBA, affecting food intake and energy balance [25].

Understanding the complex interplay between microbiota and obesity is critical for developing novel strategies to prevent and treat obesity. Targeted interventions, such as probiotics, prebiotics, synbiotics, and FMT, have shown promise in modulating the gut microbiota and potentially reducing obesity-related metabolic disturbances. However, research in this field still evolving, and numerous questions remain unanswered. Host genetics, diet, age, and environmental exposure further complicate the relationship between microbiota and obesity [26]. As our knowledge expands, harnessing the power of the gut microbiota may offer new avenues for personalized interventions and therapies to combat the obesity epidemic, ultimately improving global public health. Studies have shown that the gut microbiota is vital in regulating metabolism, energy homeostasis, and central appetite in humans [24]. It is now possible to treat metabolic disorders, such as obesity, with the microbiota.

Probiotics

Probiotics refer to “live microorganisms that confer a health benefit on the host when administered in adequate amounts.” Studies have demonstrated a link between probiotics and weight loss in animals and humans [27]. The Firmicutes to Bacteroidetes ratio increases in obese individuals because of poor or excessive food intake. Evidence suggests that probiotics and synbiotics reduce body weight through different mechanisms by facilitating energy extraction from the ingested food and increasing energy storage in the host’s adipose tissue [28]. In addition to reducing intestinal permeability, probiotics prevent the translocation of bacteria and reduce inflammation caused by lipopolysaccharides (LPS) [29]. Satiety is improved by reducing inflammation in the hypothalamus, which increases insulin sensitivity. Probiotics can modulate and maintain healthy microbiota by increasing leptin concentrations in adipose tissue, glucagon-like peptide 1 (GLP-1), and Peptide YY (PYY) levels in the intestine [30]. Several bacterial strains reduce endotoxemia, adiposity, tissue inflammation, body weight, LEP levels, and energy intake. Lactobacillus and Bifidobacterium species are the most common probiotic species; however, their impact on obesity appears to be species- and strain-specific [31].

Prebiotics

Prebiotics are “selectively fermented ingredients that promote beneficial changes in the composition and activity of the gastrointestinal microbiota.” They promote the growth of beneficial bacteria in the digestive system. Future targets against obesity may include these compounds [32]. A few examples include lactulose, inulin, fructooligosaccharides, and galactose derivatives. The most common prebiotics are oligosaccharides (e.g., inulin), fructooligosaccharides, galactooligosaccharides, and polyphenols. Using these substances as a medium can stimulate the growth of probiotics. A significant effect of prebiotic consumption is on gastrointestinal microbiota composition and metabolic activity. There are several ways in which prebiotics influence lipid metabolism, the immune system, calcium absorption, and bowel function [33]. Prebiotics increase the number of beneficial bacteria in the gastrointestinal tract, such as Bifidobacterium and Lactobacillus spp. Among their effects are antiobesity, reduced metabolic endotoxemia, decreased circulating pro-inflammatory cytokines, increased SCFA production, increased expression of tight junction proteins, and enhanced intestinal barrier function [34]. Inulin-type prebiotics promote the growth of beneficial lactobacilli and bifidobacteria in PYY responses and GLP-1 and reduce serum ghrelin levels, which may affect food intake. Patients with obesity and type 2 diabetes have fewer beneficial bacteria than healthy individuals [35]. More research is required to explain the precise mechanisms of prebiotics and obesity.

Synbiotics

A synbiotic is a combination of prebiotics and probiotics that possess synergistic effects. Synbiotics maximize prebiotic and probiotic effectiveness and maximize their beneficial effects on the host [36]. The most crucial consequence of the production of synbiotics is an increase in the survival of probiotics in the gastrointestinal tract. In addition to providing specific health benefits, synbiotics also enhance the viability of probiotic microorganisms. Synbiotics modulate metabolic activity in the intestine by developing microbiota, maintaining intestinal biostructure, and inhibiting potential pathogens in the gastrointestinal tract. Synbiotics reduce the number of undesirable metabolites by inactivating nitrosamines and carcinogenic substances in the gastrointestinal tract [37]. They also elevate SCFAs, carbon disulfide ketones, and methyl acetate levels, which may positively impact the host’s health [29].

Clinical studies in obesity

Obesity is a global pandemic today, and gut-microbial modulation is one of the areas of research for new therapeutic options in obesity. Gut microbiota is extensively studied in preclinical models. This review elaborates on the findings from human clinical trials and observational studies. The studies are summarized in Table 1.

**Table 1:** Clinical studies in obesity BMI: body mass index; FMT: fecal microbiota transplantation

Author	Key Findings
Kadooka et al. [38]	Reduced abdominal fat, potential metabolic benefits
Larsen et al. [39]	Modified microbiota, particularly Bacteroides-Prevotella-Porphyromonas
Nicolucci et al. [40]	Prebiotics led to weight loss, changed microbiota, and increased Bifidobacterium spp.
Allegretti et al. [41]	FMT altered microbiome and bile acids, no change in BMI
Chaiyasut et al. [42]	Synbiotics altered body weight and antioxidant levels

Kadooka et al. conducted a double-blind, randomized, placebo-controlled multicenter trial in 2010. The trial evaluated the probiotic *Lactobacillus gasseri* SBT2055 in adults with obese tendencies (BMI 24.2-30.7). The intervention in the trial was fermented milk with *L. gasseri*. The study significantly reduced abdominal fat and body weight by 4.6% and 3.3%, respectively [38]. In 2013, Larsen et al. conducted a trial investigating *Lactobacillus salivarius* (Ls-33) in obese adolescents. This double-blind clinical trial compared Ls-33 with placebo in 50 participants receiving either Ls-33 or placebo and assessed microbiota, SCFAs, and other parameters. The findings showed that Ls-33 significantly modified the microbiota, particularly affecting the Bacteroides-Prevotella-Porphyromonas group [39]. In 2017, Nicolucci et al. conducted another double-blind, placebo-controlled trial in Canada. In this study, the researchers included overweight children aged 7-12 years. The children were examined after 16 weeks post-intervention with prebiotics, which showed a significant reduction in z-scores for body weight (3.1%), percent body fat (2.4%), and percent trunk fat (3.8%). The results revealed that prebiotics led to weight loss, reduced body fat, and changes in gut microbiota composition, including increased Bifidobacterium spp [40]. In 2019, Allegretti et al. conducted a pilot study evaluating FMT in obese patients without comorbidity of diabetes. This double-blind study administered FMT or placebo capsules and analyzed microbiome and bile acid profiles. Although FMT did not reduce BMI, it led to microbiome changes and shifts in bile acid profiles resembling the donor’s [41]. Chaiyasut et al. conducted a randomized controlled trial in Thai obese adults. All adults with BMI >25 kg/m^2^ were included. The study had 72 participants who were given synbiotics containing Lactobacillus and Bifidobacterium for 12 weeks. Compared to placebo, there is a significant change from baseline in antioxidant parameters and body weight in the synbiotics group. However, other parameters like BMI and body fat percentage did not show much change [42].

Clinical studies in depression and anxiety

Depression and anxiety are the two common mental health disorders studied. Many clinical trials and observational studies have further expanded our understanding of gut microbiota as a potential therapeutic option. We have reviewed some of the important clinical trials and observational studies here. The studies are summarized in Table 2.

**Table 2:** Clinical studies in depression and anxiety GAD: generalized anxiety disorder; BDNF: brain-derived natriuretic factor

Author	Findings
Jiang et al. [43]	GAD patients have reduced microbial richness, and overgrowth is observed
Valles-Colomer et al. [44]	Faecalibacterium and Coprococcus relate to higher quality of life.
Chahwan et al. [45]	Probiotics reduce cognitive reactivity and affect depression metric.
Kazemi et al. [46]	Probiotics reduced depression and altered the kynurenine/tryptophan ratio.
Ramírez-Carrillo et al. [47]	Ascaris lumbricoides perturbs microbiota, linked to depression
Mason et al. [48]	Altered microbiota related to clinical presentation
Zhu J et al. [49]	Microbiota signatures found, differences detected
Kim et al. [50]	Probiotics reduce inflammation, improve mental health, and increase BDNF levels.
Schaub et al. [51]	Probiotics reduced depression and maintained microbial diversity.
Haghighat et al. [52]	Synbiotics improve depressive and anxiety symptoms and increase BDNF levels

Jiang et al. conducted a 2018 study to analyze the gut microbiome in generalized anxiety disorder (GAD) patients. The cross-sectional study compared 40 GAD patients and 36 healthy controls and found that GAD patients had reduced microbial richness, and overgrowth of bacteria, such as Escherichia-Shigella, Fusobacterium, and *Ruminococcus gnavus* was observed. These dysbiotic findings suggested a potential link between gut microbiota and GAD, highlighting the role of the gut in mental health [43]. Valles-Colomer et al. conducted a correlation study in 2019 with a large cohort to investigate the relationship between the microbiome and quality of life in depression. This study is a part of the “Flemish Gut Flora Project” with a cohort of 1050 participants. This correlation study established the relationship between specific bacterial genera, Faecalibacterium and Coprococcus, and its positive correlation with a higher quality of life. This research found a connection between the gut microbiome and mental health, suggesting that the microbiome could influence an individual’s overall well-being [44].

Chahwan et al. conducted a triple-blind placebo-controlled trial with participants experiencing depression. They performed pre- and post-comparisons with 71 participants. The study found that probiotics significantly reduced cognitive reactivity. However, evaluating the results has to be done with caution because of the high attrition rate of the study. These findings indicate that probiotics may play a role in reducing susceptibility to depression [45]. In 2019, Kazemi et al. conducted a study comparing probiotic and prebiotic supplementation in patients with major depressive disorder. This double-blind clinical trial included 110 participants with major depression. The participants received probiotics, prebiotics, or placebo for 8 weeks. The findings indicated that probiotic supplementation resulted in a significant decrease in Beck Depression Inventory(BDI) score compared with placebo and prebiotic, and there was a substantial reduction in the kynurenine/tryptophan ratio in the probiotic group [46]. In 2020, a study conducted a network analysis using Guerrero’s data to explore the relationship between gut microbiota and parasite infection *Ascaris lumbricoides* (*A. lumbricoides*). The study suggested that the presence of *A. lumbricoides* perturbs the microbiota and shows some link to depression. These findings implied that microbiota changes associated with parasitic infection could contribute to the development of depression [47].

Moreover, another case-control study by Mason et al. in 2020 focused on microbiota characterization in participants with major depression comorbid with anxiety. This study discovered a significant correlation between anhedonia scores and clostridiales in the gut. However, the study included predominantly females (82%) and had a small sample size of 60 participants. The research revealed that the altered clostridial population was related to the clinical presentation of depression. Specifically, the absence of Clostridia was linked to depression, whereas the reduction of Bacteroides was associated with anxiety [48]. Zhu et al. characterized the microbial communities in anxiety and depression screeners. The study had 69 participants from whom DNA extraction and 16s rRNA sequencing were performed. The study revealed microbiota signatures and differences in screeners. In controls, there was a predominance of Firmicutes, Bacteriodetes, and Proteobacteria. In anxiety and depression, Prevotella was dominant. This study shed light on the microbial variations in individuals with anxiety and depression symptoms [49]. Kim et al. conducted a randomized, double-blind, and placebo-controlled multicenter trial examining the impact of probiotics on cognition and mood in healthy older adults. In this trial, they chose 63 participants of age>65. They were given either *Bifidobacterium bifidum* or *longum* for 12 weeks. The gut microbiota was analyzed using 16s RNA sequencing. The findings showed that probiotics reduced inflammation-causing gut bacteria, improved mental flexibility tests and stress scores, and increased serum brain-derived natriuretic peptide (BDNF) levels to a great extent. Changes in gut microbes negatively correlated with BDNF levels. This study has shed light on the benefits of probiotic supplementation in normal geriatric populations who are more prone to developing mental health disorders [50]. Schaub et al. conducted a randomized controlled trial in 2022 and examined the effects of probiotic supplementation on depressive symptoms in patients with major depressive disorder. Patients received a multi-strain probiotic supplement or placebo for 31 days. The findings showed that the probiotic group had decreased HAM-D scores, maintained microbial diversity, and increased Lactobacillus abundance. Reduced putamen activation in response to neutral faces is observed. However, evaluating the results has to be done cautiously because of the small sample size [51]. Haghighat et al. assessed the effect of synbiotic supplementation in hemodialysis patients with anxiety. In this randomized controlled trial, a combination of Lactobacillus and Bifidobacterium was given for 12 weeks. There is a significant reduction in anxiety and depressive symptoms with an increase in BDNF levels. However, depressive symptoms were evaluated only in a sub-group of patients [52].

## Conclusions

Gut-microbial modulation is a relatively new and emerging therapy area in various diseases. Today’s research has revealed gut microbiota’s role in physiological and homeostatic functions. Numerous preclinical studies have shown promising evidence for the links between gut microbiota and pathological conditions. Besides obesity, the GBA is studied in neurodegenerative disorders, inflammatory bowel disease, and many other conditions. Our review has extensively reviewed the translation potential of gut-microbial modulators. Various exploratory clinical trials and cross-sectional studies have shown preliminary efficacy. However, the treatment modality in the form of FMT is extensively evaluated in only a single trial, which we have reviewed. Most trials have evaluated the efficacy of combining different modalities of gut-microbial modulation, further complicating the exact inference of efficacy about individual compounds. Small sample sizes are the limitations of all the studies we have reviewed. Large therapeutic confirmatory trials will be required to generate evidence of effectiveness in clinical settings.

## References

[1] Ghaffari P, Shoaie S, Nielsen LK (2022). Irritable bowel syndrome and microbiome; switching from conventional diagnosis and therapies to personalized interventions. J Transl Med.

[2] Wang JW, Kuo CH, Kuo FC (2019). Fecal microbiota transplantation: Review and update. J Formos Med Assoc.

[3] Magne F, Gotteland M, Gauthier L, Zazueta A, Pesoa S, Navarrete P, Balamurugan R (2020). The firmicutes/bacteroidetes ratio: A relevant marker of gut dysbiosis in obese patients?. Nutrients.

[4] Gomes AC, Hoffmann C, Mota JF (2018). The human gut microbiota: Metabolism and perspective in obesity. Gut Microbes.

[5] Anlauf M, Schäfer MK, Eiden L, Weihe E (2003). Chemical coding of the human gastrointestinal nervous system: cholinergic, VIPergic, and catecholaminergic phenotypes. J Comp Neurol.

[6] Schemann M, Neunlist M (2004). The human enteric nervous system. Neurogastroenterol Motil.

[7] Sgritta M, Dooling SW, Buffington SA, Momin EN, Francis MB, Britton RA, Costa-Mattioli M (2019). Mechanisms underlying microbial-mediated changes in social behavior in mouse models of autism spectrum disorder. Neuron.

[8] Cryan JF, O'Riordan KJ, Cowan CS (2019). The microbiota-gut-brain axis. Physiol Rev.

[9] Lee A, Lee JY, Jung SW (2023). Brain-gut-microbiota axis. (Article in Korean). Korean J Gastroenterol.

[10] Martin CR, Osadchiy V, Kalani A, Mayer EA (2018). The brain-gut-microbiome axis. Cell Mol Gastroenterol Hepatol.

[11] Wang HX, Wang YP (2016). Gut microbiota-brain axis. Chin Med J (Engl).

[12] Perler BK, Friedman ES, Wu GD (2023). The role of the gut microbiota in the relationship between diet and human health. Annu Rev Physiol.

[13] (2023). The Gut Microbiome and Drug Addiction: An Emerging Link. https://asm.org/articles/2023/april/the-gut-microbiome-and-drug-addiction-an-emerging.

[14] Agustí A, García-Pardo MP, López-Almela I, Campillo I, Maes M, Romaní-Pérez M, Sanz Y (2018). Interplay between the gut-brain axis, obesity and cognitive function. Front Neurosci.

[15] Round JL, Mazmanian SK (2009). The gut microbiota shapes intestinal immune responses during health and disease. Nat Rev Immunol.

[16] Rooks MG, Garrett WS (2016). Gut microbiota, metabolites and host immunity. Nat Rev Immunol.

[17] Cussotto S, Sandhu KV, Dinan TG, Cryan JF (2018). The neuroendocrinology of the microbiota-gut-brain axis: A behavioural perspective. Front Neuroendocrinol.

[18] De Vadder F, Kovatcheva-Datchary P, Goncalves D (2014). Microbiota-generated metabolites promote metabolic benefits via gut-brain neural circuits. Cell.

[19] Sudo N, Chida Y, Aiba Y (2004). Postnatal microbial colonization programs the hypothalamic-pituitary-adrenal system for stress response in mice. J Physiol.

[20] Goehler LE, Gaykema RP, Opitz N, Reddaway R, Badr N, Lyte M (2005). Activation in vagal afferents and central autonomic pathways: Early responses to intestinal infection with Campylobacter jejuni. Brain Behav Immun.

[21] NCD Risk Factor Collaboration (NCD-RisC) (2016). Trends in adult body-mass index in 200 countries from 1975 to 2014: a pooled analysis of 1698 population-based measurement studies with 19·2 million participants. Lancet.

[22] Asadi A, Shadab Mehr N, Mohamadi MH, Shokri F, Heidary M, Sadeghifard N, Khoshnood S (2022). Obesity and gut-microbiota-brain axis: A narrative review. J Clin Lab Anal.

[23] Aoun A, Darwish F, Hamod N (2020). The influence of the gut microbiome on obesity in adults and the role of probiotics, prebiotics, and synbiotics for weight loss. (Article in Korean). Prev Nutr Food Sci.

[24] Leeuwendaal NK, Cryan JF, Schellekens H (2021). Gut peptides and the microbiome: Focus on ghrelin. Curr Opin Endocrinol Diabetes Obes.

[25] Muscogiuri G, Cantone E, Cassarano S, Tuccinardi D, Barrea L, Savastano S, Colao A (2019). Gut microbiota: A new path to treat obesity. Int J Obes Suppl.

[26] Chakraborti CK (2015). New-found link between microbiota and obesity. World J Gastrointest Pathophysiol.

[27] So D, Whelan K, Rossi M (2018). Dietary fiber intervention on gut microbiota composition in healthy adults: A systematic review and meta-analysis. Am J Clin Nutr.

[28] Torres S, Fabersani E, Marquez A, Gauffin-Cano P (2019). Adipose tissue inflammation and metabolic syndrome. The proactive role of probiotics. Eur J Nutr.

[29] Álvarez-Arraño V, Martín-Peláez S (2021). Effects of probiotics and synbiotics on weight loss in subjects with overweight or obesity: A systematic review. Nutrients.

[30] Wu T, Wang G, Xiong Z (2022). Probiotics interact with lipids metabolism and affect gut health. Front Nutr.

[31] Abenavoli L, Scarpellini E, Colica C (2019). Gut microbiota and obesity: A role for probiotics. Nutrients.

[32] Cerdó T, García-Santos JA, G Bermúdez M, Campoy C (2019). The role of probiotics and prebiotics in the prevention and treatment of obesity. Nutrients.

[33] John GK, Mullin GE (2016). The gut microbiome and obesity. Curr Oncol Rep.

[34] Holscher HD (2017). Dietary fiber and prebiotics and the gastrointestinal microbiota. Gut Microbes.

[35] Iatcu CO, Steen A, Covasa M (2021). Gut microbiota and complications of type-2 diabetes. Nutrients.

[36] Cencic A, Chingwaru W (2010). The role of functional foods, nutraceuticals, and food supplements in intestinal health. Nutrients.

[37] Dos Santos Cruz BC, da Silva Duarte V, Sousa Dias R, Ladeira Bernardes A, de Paula SO, de Luces Fortes Ferreira CL, do Carmo Gouveia Peluzio M (2022). Synbiotic modulates intestinal microbiota metabolic pathways and inhibits DMH-induced colon tumorigenesis through c-myc and PCNA suppression. Food Res Int.

[38] Kadooka Y, Sato M, Imaizumi K (2010). Regulation of abdominal adiposity by probiotics (Lactobacillus gasseri SBT2055) in adults with obese tendencies in a randomized controlled trial. Eur J Clin Nutr.

[39] Larsen N, Vogensen FK, Gøbel RJ, Michaelsen KF, Forssten SD, Lahtinen SJ, Jakobsen M (2013). Effect of Lactobacillus salivarius Ls-33 on fecal microbiota in obese adolescents. Clin Nutr.

[40] Nicolucci AC, Hume MP, Martínez I, Mayengbam S, Walter J, Reimer RA (2017). Prebiotics reduce body fat and alter intestinal microbiota in children who are overweight or with obesity. Gastroenterology.

[41] Allegretti JR, Kassam Z, Mullish BH (2020). Effects of fecal microbiota transplantation with oral capsules in obese patients. Clin Gastroenterol Hepatol.

[42] Chaiyasut C, Sivamaruthi BS, Kesika P (2021). Synbiotic supplementation improves obesity index and metabolic biomarkers in Thai obese adults: A randomized clinical trial. Foods.

[43] Jiang HY, Zhang X, Yu ZH, Zhang Z, Deng M, Zhao JH, Ruan B (2018). Altered gut microbiota profile in patients with generalized anxiety disorder. J Psychiatr Res.

[44] Valles-Colomer M, Falony G, Darzi Y (2019). The neuroactive potential of the human gut microbiota in quality of life and depression. Nat Microbiol.

[45] Chahwan B, Kwan S, Isik A, van Hemert S, Burke C, Roberts L (2019). Gut feelings: A randomised, triple-blind, placebo-controlled trial of probiotics for depressive symptoms. J Affect Disord.

[46] Kazemi A, Noorbala AA, Azam K, Eskandari MH, Djafarian K (2019). Effect of probiotic and prebiotic vs placebo on psychological outcomes in patients with major depressive disorder: A randomized clinical trial. Clin Nutr.

[47] Ramírez-Carrillo E, Gaona O, Nieto J (2020). Disturbance in human gut microbiota networks by parasites and its implications in the incidence of depression. Sci Rep.

[48] Mason BL, Li Q, Minhajuddin A (2020). Reduced anti-inflammatory gut microbiota are associated with depression and anhedonia. J Affect Disord.

[49] Zhu J, Li M, Shao D, Ma S, Wei W (2021). Altered fecal microbiota signatures in patients with anxiety and depression in the gastrointestinal cancer screening: A case-control study. Front Psychiatry.

[50] Kim CS, Cha L, Sim M, Jung S, Chun WY, Baik HW, Shin DM (2021). Probiotic supplementation improves cognitive function and mood with changes in gut microbiota in community-dwelling older adults: A randomized, double-blind, placebo-controlled, multicenter trial. J Gerontol A Biol Sci Med Sci.

[51] Schaub AC, Schneider E, Vazquez-Castellanos JF (2022). Clinical, gut microbial and neural effects of a probiotic add-on therapy in depressed patients: a randomized controlled trial. Transl Psychiatry.

[52] Haghighat N, Rajabi S, Mohammadshahi M (2021). Effect of synbiotic and probiotic supplementation on serum brain-derived neurotrophic factor level, depression and anxiety symptoms in hemodialysis patients: a randomized, double-blinded, clinical trial. Nutr Neurosci.

